# Sexual dimorphic gene expression profile of perirenal adipose tissue in ovine fetuses with growth restriction

**DOI:** 10.3389/fphys.2023.1179288

**Published:** 2023-08-04

**Authors:** Jack Blomberg, Rosa I. Luna Ramirez, Dipali Goyal, Sean W. Limesand, Ravi Goyal

**Affiliations:** School of Animal and Comparative Biomedical Sciences, College of Agriculture and Life Sciences, University of Arizona, Tucson, AZ, United States

**Keywords:** adipose, IUGR, placental insufficiency, fetal growth restriction, RNAseq, sexually dimorphic, epigenetics

## Abstract

Worldwide, fetal growth restriction (FGR) affects 7%–10% of pregnancies, or roughly 20.5 million infants, each year. FGR increases not only neonatal mortality and morbidity but also the risk of obesity in later life. Currently, the molecular mechanisms by which FGR “programs” an obese phenotype are not well understood. Studies demonstrate that FGR females are more prone to obesity compared to males; however, the molecular mechanisms that lead to the sexually dimorphic programming of FGR are not known. Thus, we hypothesized that FGR leads to the sexually dimorphic programming of preadipocytes and reduces their ability to differentiate into mature adipocytes. To test the hypothesis, we utilized a maternal hyperthermia-induced placental insufficiency to restrict fetal growth in sheep. We collected perirenal adipose tissue from near-term (∼140 days gestation) male and female FGR and normal-weight fetal lambs (*N* = 4 to 5 in each group), examined the preadipocytes’ differentiation potential, and identified differential mRNA transcript expression in perirenal adipose tissue. Male FGR fetuses have a lower cellular density (nuclei number/unit area) compared to control male fetuses. However, no difference was observed in female FGR fetuses compared to control female fetuses. In addition, the ability of preadipocytes to differentiate into mature adipocytes with fat accumulation was impaired in male FGR fetuses, but this was not observed in female FGR fetuses. Finally, we examined the genes and pathways involved in the sexually dimorphic programming of obesity by FGR. On enrichment of differentially expressed genes in males compared to females, the Thermogenesis KEGG Pathway was downregulated, and the Metabolic and Steroid Biosynthesis KEGG pathways were upregulated. On enrichment of differentially expressed genes in male FGR compared to male control, the Steroid Biosynthesis KEGG Pathway was downregulated, and the PPAR Signaling KEGG pathway was upregulated. No pathways were altered in females in response to growth restriction in perirenal adipose tissue. Thus, the present study demonstrates a sexually dimorphic program in response to growth restriction in sheep fetal perirenal adipose tissue.

## Introduction

Obesity has emerged as a major health crisis in the United States and worldwide. According to the WHO, the prevalence of obesity has tripled since 1975 (WHO, 2020). Based on the CDC data, the prevalence of obesity was 42.4% in 2017 and 2018. Financially, obesity plays a large part in higher medical costs, decreased productivity, and increased absence from work (Tremmel et al., 2017). With healthcare costs rising every day, the cost of excess healthcare expenditures takes an enormous amount of toll on individuals. In 2014, globally, the economic impact of obesity was $2.0 trillion US dollars (Tremmel et al., 2017)^.^ Additionally, obesity significantly reduces life span and quality of life. It is shown that obesity can decrease life expectancy by as much as 5–10 years (Fruh, 2017). Obesity is related to disorders such as coronary artery disease, arthritis, and stroke. Obesity also has a considerable impact on mental health conditions like low self-esteem, mood disorder, and eating disorders (Djalalinia et al., 2015). Overall, obesity is immensely impactful on society and requires urgent attention and investigation.

Human epidemiological studies demonstrate that FGR is a major risk factor for obesity later in life (Barker, 1994). This has led to the concept of developmental origins of health and disease (DOHaD) and “fetal programming” of obesity with associated metabolic disorders (Hales and Barker, 2001). Several studies support that FGR predisposes individuals to a significantly higher risk of developing obesity (Hanson and Gluckman, 2008; Goyal and Longo, 2013). The thrifty phenotype hypothesis explains the mechanism of fetal programming that the *in utero* environment of reduced nutrient availability prepares the fetus for the predicted outside environment of nutrient scarcity after birth (Hales and Barker, 2001). Several studies support that a fetus is “programmed” to live under thrifty conditions (Ozanne et al., 2004; Hochberg et al., 2011; Goyal and Longo, 2013; Yajnik, 2014; Ducsay et al., 2018; Goyal et al., 2019). If, postnatally, there is adequate nutrition available to the newborn, it starts accumulating excess nutrition as fat for future nutritional starvation. This leads to rapid catch-up growth, which is a risk factor for metabolic syndrome (Goyal and Longo, 2013; Crume et al., 2014). Mechanisms of such programming are not entirely understood (Ducsay et al., 2018; Goyal et al., 2019). Nonetheless, it is well established that FGR predisposes to obese phenotype (Longo et al., 2014a; Longo et al., 2014b; Longo et al., 2014c; Goyal et al., 2019). Obesity is a very complex process and depends on the precise regulation of gene expression (Albuquerque et al., 2017). This complexity is in part because of the complex composition of adipose tissue, which contains several different cell types such as white adipocytes, which can accumulate fat, and brown adipocytes, which efficiently convert energy into heat, an intermediate phenotype known as beige adipocytes, and cells with stem cells like property (known as preadipocytes, vascular-stromal fraction, or adipose tissue-derived stem cells). These preadipocytes are very plastic cell types (Gimble et al., 2007). For instance, it can be differentiated into all three lineages—endodermal (Mohamad Buang et al., 2012), mesodermal (Joo et al., 2017), and ectodermal origins (Huang et al., 2007). However, the FGR-mediated programming of adipocytes is not well investigated and creates a significant knowledge gap in our current understanding.

There are several animal models to create FGR. Our group has used various methods to generate FGR, including maternal protein malnutrition (Goyal and Longo, 2013), hypoxic stress (Goyal et al., 2011), high altitude acclimatization (Goyal and Longo, 2014), and increasing ambient temperature during gestation to 40°C (104°F) (Camacho et al., 2017; Yates et al., 2019). All these methods produce significant FGR. However, based on our studies, ambient hyperthermia produces significant placental insufficiency and closely mimics human FGR (Yates et al., 2011). The ambient hyperthermia model was based on the observation that lambs born in the summer have lower birth weights and higher mortality rates than lambs born in cooler seasons (Shelton et al., 2014).

In the mouse, we have demonstrated that FGR pups are more prone to obesity and hypertension in a sexually dimorphic manner (Goyal and Longo, 2013). Others have also demonstrated that FGR babies are at significant risk for excessive fat deposition in adipose tissues and rapid catch-up growth in a sexually dimorphic manner (Hales and Barker, 2001; Tchoukalova et al., 2014; Dearden et al., 2018). Specifically, FGR females are prone to develop greater obesity compared to males in later life. However, in mice, the metabolic rate is about seven times larger than humans and is 2,000–3,000 times smaller than humans (Patrick et al., 2008). Also, unlike large animals, rodents are born with immature BAT, which matures only postnatally and is largely retained throughout life (Gemmell et al., 1972; Cannon and Nedergaard, 2004). Additionally, in sheep, the deposition of adipose tissue and the development of key organ systems regulating metabolism and energy balance occurs before birth in this species, as in humans. Of note, human visceral fat contains high levels of immune cells, which are not present in mice, and mice visceral fat functions chiefly as a cushion for organs and storage for lipids (Chusyd et al., 2016). Thus, several recent studies indicate that large animal models, such as sheep, are better for studying adipogenesis (Patrick et al., 2008; Morrison et al., 2018; Borgeson et al., 2022).

Thus, we hypothesized that FGR leads to the sexually dimorphic programming of preadipocytes and reduces their ability to differentiate into mature adipocytes.

## Methods

All animal studies were reviewed and approved by the Institutional Animal Care and Use Committee (IACUC) of the University of Arizona. Crossbred Columbia-Rambouillet ewes carrying singleton pregnancies were used for the present study. All studies were conducted on the four study groups—near-term control male fetuses (MC), near-term control female fetuses (FC), near-term FGR male fetuses (MFGR), and near-term FGR female fetuses (FFGR).

### FGR animal model

Pregnant ewes were exposed to high ambient temperatures to produce maternal hyperthermia that causes progressive placental insufficiency and fetal growth restriction (Bell et al., 1987; Limesand et al., 2018). In this model of FGR, pregnant ewes are exposed to elevated ambient temperatures (40°C for 12 h; 35°C for 12 h; dew point 22°C) from 38 ± 1 to 87 ± 1 day of gestation (total gestation in sheep is ∼149 days). Control fetuses are from ewes maintained at 22°C ± 1°C and pair-fed to the average *ad libitum* feed intake of the hyperthermic group (Bell et al., 1987; Galan et al., 1999; Regnault et al., 2002; Chen et al., 2010; Leos et al., 2010; Limesand and Rozance, 2017). All ewes are given *ad libitum* access to water and salt. Following physiological measurements previously reported (Pendleton et al., 2020), the ewe and fetus were killed humanely (Euthasol; Virbac Animal Health).

### Adipose tissue collection

Perirenal adipose tissue from four experimental groups was collected at term (∼140 days of gestation) to examine morphological differences and changes in gene expression. Perirenal adipose tissue is a visceral subtype with well-defined boundaries and is covered with a perirenal fascia (Gerota’s fascia), which allows for complete dissection and weight measurements. After weighing the adipose tissue, a portion of fresh perirenal adipose tissue was used to isolate preadipocytes, a slice was fixed in 4% paraformaldehyde, and the rest was snap-frozen in liquid nitrogen and stored at −80° for downstream RNAseq analysis (Cole et al., 2009; Chen et al., 2010).

### Cell density in adipose tissue

Cell Density was examined by staining the section with Hoechst live-cell stains. The blue nuclei stained were counted using ImageJ software. Five animals were used in each group.

### Triglyceride content assay

Triglyceride content of the perirenal adipose tissue in the four study groups (four animals in each group) was determined by the Triglyceride-Glo™ Assay (Promega, Inc; Cat #J3161) following manufacturer’s instructions. The Assay detects triglyceride levels by measuring glycerol that is released from triglycerides in an enzymatic reaction with a lipase: one molecule of glycerol per molecule of triglyceride. Glycerol is measured in a coupled reaction scheme that links the production of NADH to the activation of a proluciferin that produces light with luciferase. The amount of triglyceride is determined from the difference of glycerol measured in the absence (free glycerol) and presence (total glycerol) of lipase. The assay measures triglyceride by conversion to glycerol and fatty acids by lipase. Briefly, 50 mg of tissue was homogenized with liquid nitrogen and dissolved in the glycerol lysis buffer with and without lipase. Enzymes and substrate were added as per the manufacturer’s instruction, and the luminescence was measured by a luminometer (Biotek Inc.)

### Histological examination by H & E staining

H & E staining was conducted using Harris hematoxylin solution and Eosin Y Solution on 10-micron sections prepared from freshly frozen perirenal adipose tissue from the four groups following the standard process.

### Preadipocyte isolation

Preadipocytes were isolated using a published protocol (Lee and Fried, 2014). Briefly, 500 mg of adipose tissue was minced in a sterile Petri dish, and 2 mL of 5 mg/mL collagenase in sterile PBS was added to the minced tube and incubated in an orbital shaker for 1 h at 37°C at 400 RPM. The tissue was triturated after every 15 min. Following the incubation, the media at the bottom of the tube was removed and filtered with a 40-micron filter. The filtrate was centrifuged at 1,000 *g* for 5 min. The pellet was mixed in RBC lysis buffer (Fisher Scientific, Cat # 501129743) and incubated at room temperature for 10 min. The cells were re-pelleted by centrifugation at 500 *g* for 5 min. The pelleted cells were mixed in DMEM with 10% FBS and 1% Pen-Strep to the culture at 37 C in 5% CO_2_ and air. The cells were washed the next day with sterile PBS, and the cells which adhered to the plastic surface of the cell culture flask were passaged further.

### Preadipocyte differentiation

Adipogenic differentiation was initiated by culturing the preadipocytes in adipogenesis initiation media (DMEM with 10% FBS, 1 µM insulin, 1 µM Dexamethasone, and 500 µM 3-isobutyl-1-methylxanthine (IBMX) and 1% penicillin-streptomycin) for 7 days. Following this, adipogenesis differentiation media (DMEM with 10% FBS, 1 µM insulin, 1 µM Dexamethasone, indomethacin 50 μM, rosiglitazone 20 μM, and 500 µM IBMX was added for the next 9 days. After 16 days of differentiation, Oil Red Staining was conducted by fixing the cells in calcium fixative and oil red staining solution as published (Zhu et al., 2013). Preadipocytes from four different animals were used in each group.

### Whole transcriptomic analysis

To determine the mechanistic pathways involved in this sexually dimorphic programming of adipocytes, we conducted RNAseq on the perirenal adipose tissue from the four groups (four animals were used in each group) following standard protocol. Briefly, the stored adipose tissue was thawed in Trizol and homogenized using 3 pulses, 4–6 s each. Then, 0.2 mL of chloroform was added per 1 mL of Trizol (0.1 mL per sample). Samples were centrifuged for 15 min at 14,000 x g (4°C), and the mixture separated into a lower red phenol-chloroform, interphase, and a colorless upper aqueous solution. The aqueous phase containing RNA was transferred to a new tube very carefully to ensure proteins and other cell components did not contaminate the RNA. Then, 250 µL of isopropanol was added to the aqueous phase (0.5 mL/mL of Trizol) and incubated for 10 min. The sample was centrifuged for 10 min at 14,000 x g (4°C), and the RNA precipitate formed a white gel-like pellet at the bottom of the tube. The supernatant was discarded, and the sample was vortexed briefly before centrifugation for 5 min at 8,000 x g (4°C). The supernatant was discarded, and the RNA pellet was air-dried for 10 min. Pellets were resuspended in 50 µL of RNase-free water. The RNA was further purified by Zymo Purelink RNA columns. The obtained RNA samples were measured for quantity (ng/mL) and purity on Nanodrop and Qubit (broad-range) according to a 260/280 nucleic acid absorbance ratio before sending the University of Arizona Genetics Core Facility for sequencing, where RNA Samples were further checked for quality and quantity with an Advanced Analytics Fragment Analyzer (High Sensitivity RNA Analysis Kit—Catalog # DNF-491) and quantity with determined with a Qubit RNA quantification kit (Qubit® RNA HS Assay Kit—Catalog #Q32852).

Once quality and quantity were validated, a library was constructed from samples using a Swift RNA Library Kit—(Catalog #R1024) and Swift Dual Combinatorial Indexing Kit—(Catalog #X8096). Upon constructing the library, the average fragment size in the library was determined with the Advanced Analytics Fragment Analyzer with the High Sensitivity NGS Analysis Kit—(Catalog # DNF-486). Quantity was evaluated with an Illumina Universal Adaptor-specific qPCR kit, the Kapa Library Quantification Kit for Illumina NGS—(Catalog # KK4824).

After completing the final library QC, samples were equimolar-pooled and clustered for sequencing on the NextSeq500 machine. The sequencing run was performed using Illumina NextSeq500 run chemistry (NextSeq 500/550 High Output v2 kit 150 cycles—Catalog FC-404–2002).

### RNAseq data analysis

Sequence data quality was validated for RNAseq analysis using FastQC Version 0.11.9. Sequences with average Phred scores below 34 were discarded. Fully annotated genome indices were generated for sheep (Oar_rambouillet_v1.0), cow (ARS-UCD1.2), mouse (GRCm39), and human (GRCh38. p13) using ENSEMBL (v1.0.103) and aligned with the sequencing data using Salmon (Patro et al., 2017). Integrated differential expression and pathway analysis were conducted with iDep.96 web-based applications (Ge et al., 2018). During preprocessing, the genes with less than 20 counts per million in 3 or more libraries were discarded from further processing, and counts were normalized using EdgeR: log2(CPM + c) transformation. The distribution of the transformed data box and density plots are provided in Supplementary Figure S1. Enrichment analysis for differentially expressed genes was conducted using a False Discovery Rate (FDR) cutoff of 0.05 and a minimum fold change of 1.5. Genes altered were matched with the known Kyoto Encyclopedia of Genes and Genomes (KEGG) pathways (Kanehisa and Goto, 2000), and gene set enrichment analysis (GSEA) was conducted as published (Subramanian et al., 2005). Generally Applicable Gene-set Enrichment (GAGE) was also conducted to determine the biological relevance of the regulatory mechanism (Luo et al., 2009).

### Statistical analysis

The four groups, MC, MFGR, FC, and FFGR, were compared using two-way ANOVA with Sex as a row factor and growth restriction as a column factor. Multiple comparisons were conducted using Tukey Test (Lee and Lee, 2018). All the error bars represent the mean ± standard error of the mean (SEM).

## Results

### Effect of ambient hyperthermia on fetal and perirenal adipose weight

**FIGURE 1:**
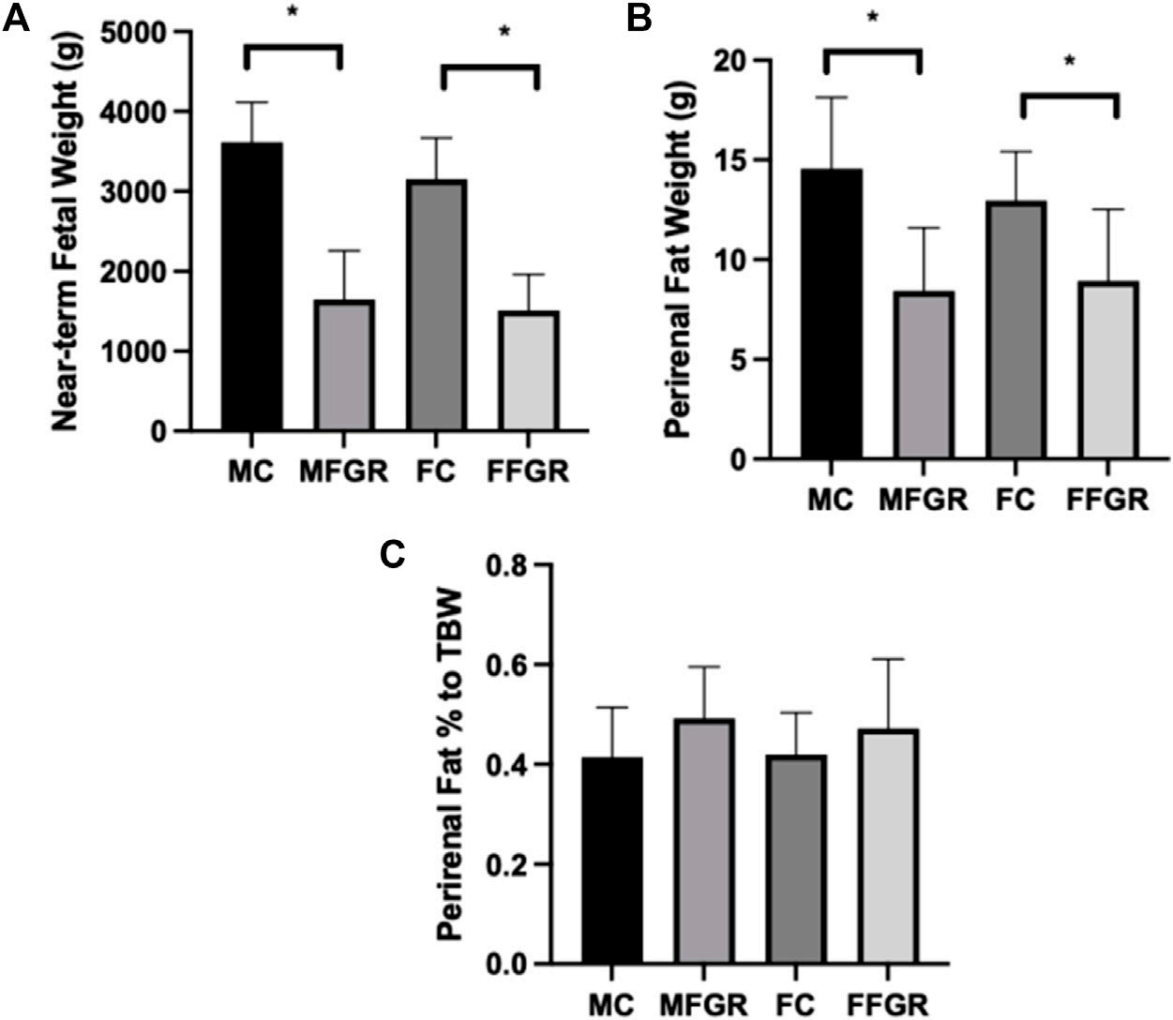
Bar graphs demonstrate: **(A)** Body weights, **(B)** Perirenal fat weights, and **(C)** Perirenal fat weight as a % of total body weight. *N* = 5 in each group, and * denotes *p* < 0.05. MC, Male control; FC, Female Control; MFGR, Growth Restricted Male Fetus; FFGR, Growth Restricted Female Fetus.

Maternal hyperthermia reduced the body weight of both male and female fetuses (Figure 1A). The average perirenal adipose tissue weight was less in both FGR males and females compared to the control counterparts (Figure 1B). However, when perirenal adipose tissue was normalized to total fetal body weight (TBW), there was no difference between FGR and control groups in both sexes (Figure 1C).

#### Cellular density, morphology, and triglyceride accumulation in perirenal adipose tissue

**FIGURE 2:**
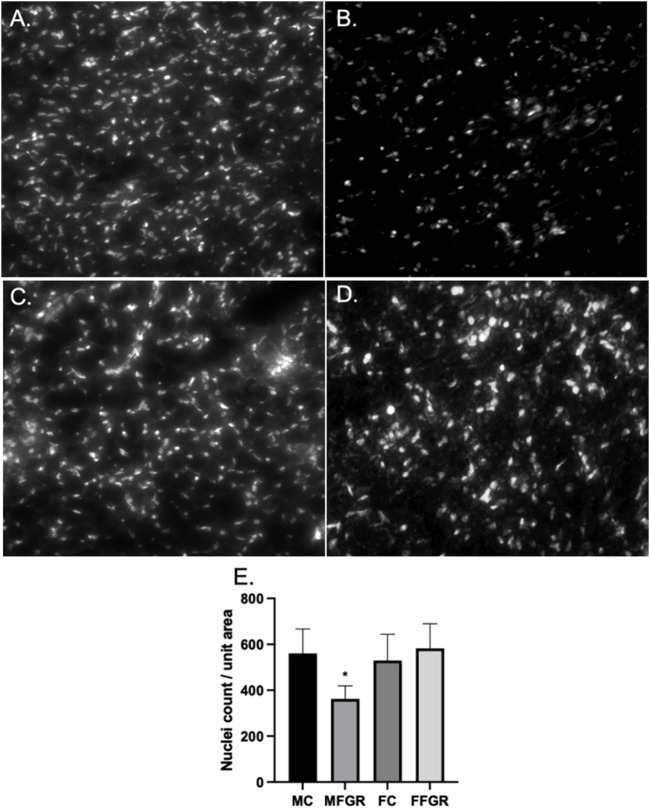
Demonstrates the exemplary photographs of nuclei staining by Hoechst live stain in MC **(A)**, MFGR **(B)**, FC **(C)**, and FFGR **(D)**. Panel **(E)** demonstrates the nuclei count per unit area of perirenal fat 10-micron slices from control and growth-restricted ovine near-term fetuses. *N* = 5 in each group, and * denotes *p* < 0.05. MC, Male control; FC, Female Control; MFGR, Growth Restricted Male Fetus; FFGR, Growth Restricted Female Fetus.

**FIGURE 3:**
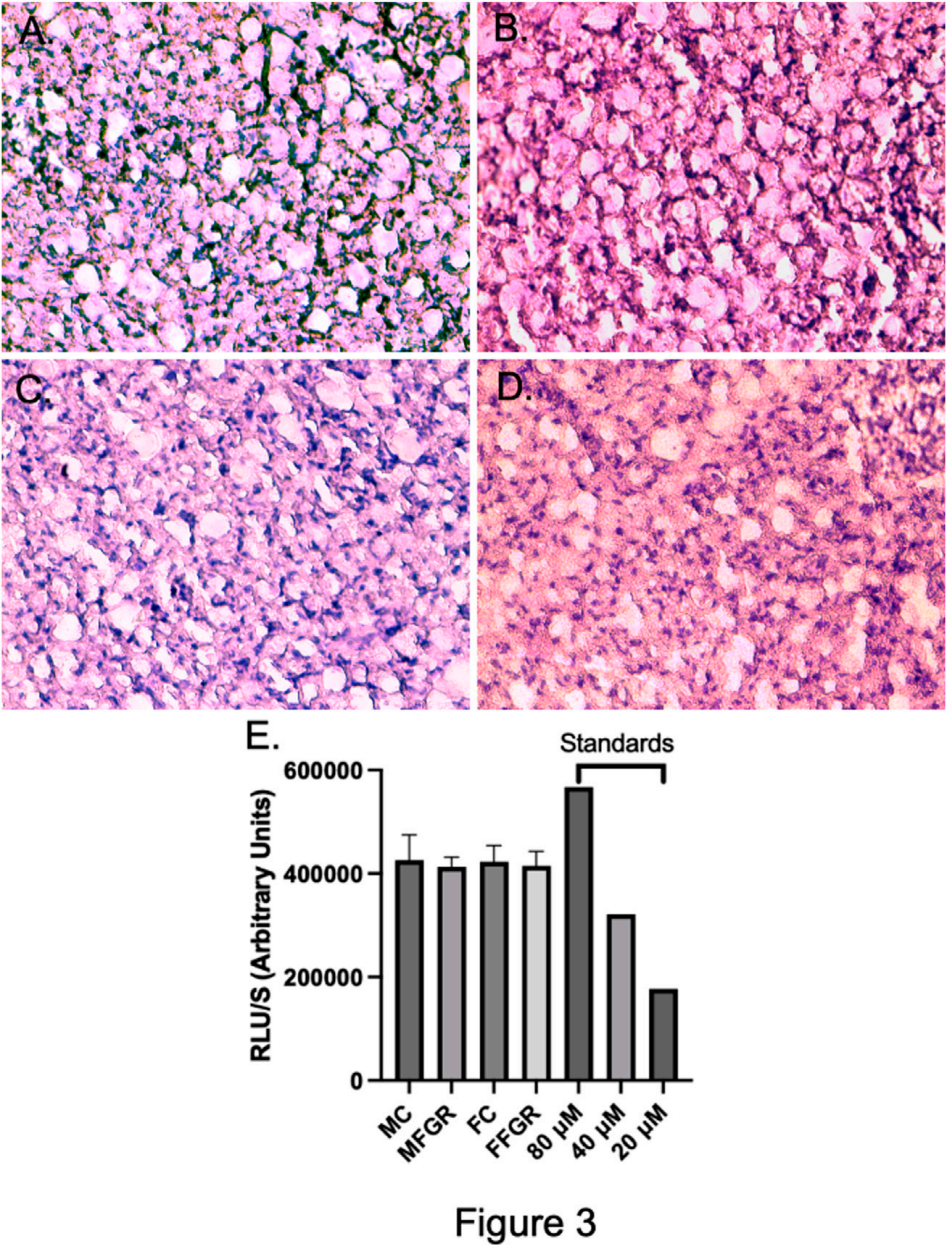
Demonstrates the exemplary H & E staining of the 10-micron slices from MC **(A)**, MFGR **(B)**, FC **(C)**, and FFGR **(D)**. Panel **(E)** demonstrates the Triglyceride Accumulation Assay in the perirenal adipose tissue from the four groups of animals. *N* = 4 in each group.

The results demonstrate a significantly lower nuclei number/unit area in FGR males (Figure 2B) compared to all other groups (Figures 2A, C–E). However, there was not much difference in the gross histology of the perirenal adipose tissue from the four groups (Figures 3A–D). Also, there was no difference in triglyceride accumulation (Figure 3E).

### Sexually dimorphic programming of preadipocyte differentiation

**FIGURE 4:**
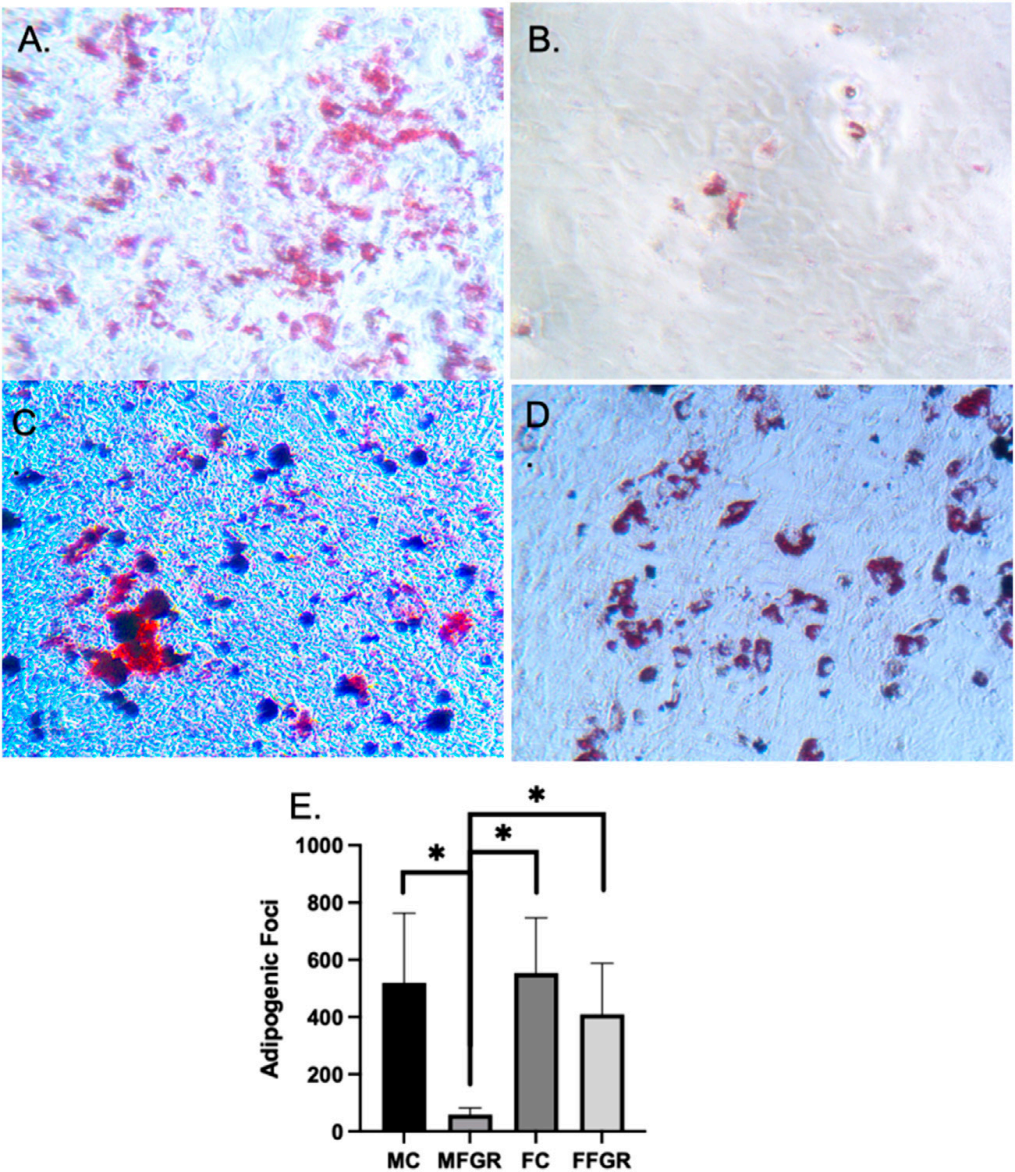
Demonstrates oil red staining of fat droplets following adipogenic differentiation of preadipocytes. **(A)** MC group. **(B)** MFGR group. **(C)** FC group. **(D)** FFGR group. **(E)** Bar graph of droplet counts. *N* = 5. * Denotes *p* < 0.05.

The results (Figure 4) demonstrate that the preadipocyte differentiation into mature adipocytes containing lipid droplets as stained by oil red stain was significantly reduced in MFGR (Figure 4B) compared to all other groups (Figures 4A, C, D). There was no significant effect of sex on the differentiation ability of preadipocytes into mature adipocytes in control males and females (Figure 4E).

### RNAseq analysis of the perirenal adipose tissue

**FIGURE 5:**
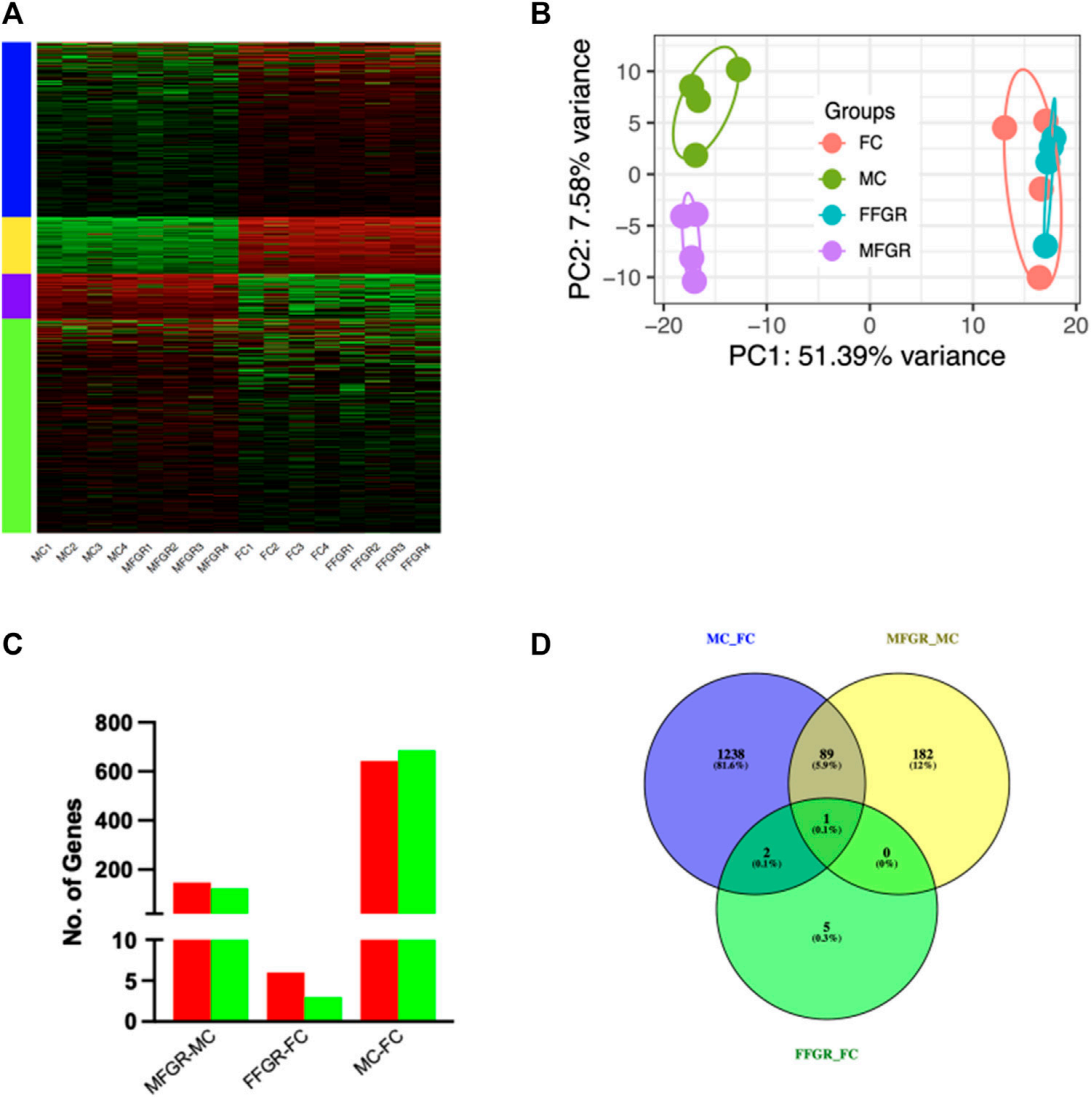
Sexually dimorphic clustering of gene expression in the four groups. **(A)** Hierarchical clustering of the differentially expressed genes in perirenal adipose tissue from male and female sheep fetuses. **(B)** PCA plot of the differential gene expression in the four study groups. **(C)** The bar graph demonstrates upregulated (red) and downregulated (green) genes by comparing the MFGR with MC, FFGR with FC, and MC with FC. **(D)** Venn diagram demonstrates the number of genes that overlapped with sex and growth restriction variables.

**FIGURE 6:**
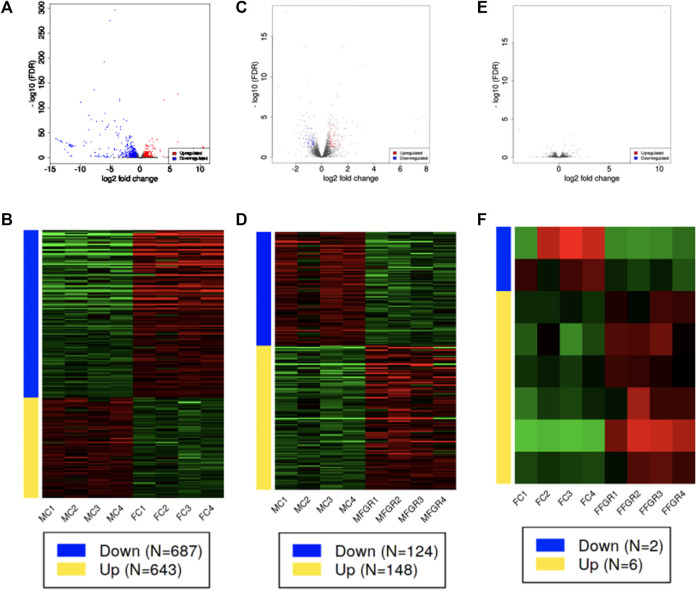
Demonstrates differential gene expression in the three comparisons (MC vs. FC; MFGR vs. MC; and FFGR vs. FC). Panels **(A, C, E)** are the volcano plots demonstrating −log10 FDR (*Y*-axis) and log2 fold change (*X*-axis) of the differentially expressed genes in the three comparative groups. Panels **(B, D, F)** demonstrate the hierarchical clustering of the differentially regulated genes in each comparative group.

Results demonstrate a strong effect of sex on gene clustering (Figure 5A). The four different colors of the sidebar demonstrated in Figure 5A denote the four different clusters of differentially regulated genes. The principal component analysis (PCA) demonstrates that growth restriction did not have much effect on gene expression in female fetuses (Female groups—FC and FFGR are clustered together on the PCA) (Figure 5B). In contrast, in male fetuses, the gene groups (MC and MFGR) clustered separately following growth restriction (Figure 5B). There were few differentially expressed genes in females compared to males with growth restriction (Figures 5C, D). A total of 1330 gene transcripts were differentially regulated (FDR <0.05 and FC > 1.5) in male and female perirenal adipose tissue (Figures 5C, D). Of these, 643 were upregulated, and 687 were downregulated in female fetus perirenal adipose tissue as compared to adipose tissue from male fetuses (Figures 6A, B). Similarly, 148 genes were upregulated, and 124 were downregulated in MFGR as compared to MC (Figures 6C, D). Surprisingly, in females, only 6 genes were upregulated and 2 downregulated in FFGR perirenal adipose tissue as compared to FC (Figures 6E, F). On comparative analysis (Figure 5D), there was only 1 gene commonly altered in response to growth restriction in visceral adipose from both males and females (Novel gene ENSOARG00020003728). The complete list of differentially regulated genes in males as compared to females is provided in Supplementary Figure S1–S3. Also, the heat maps (Figure 5A) and PCA (Figure 5B) demonstrate a separate clustering of males and females, indicating a strong effect of biological sex on gene expression in perirenal adipose tissue during fetal life. The raw RNAseq files and analyzed data sets are deposited in the GEO repository accession no. GSE235935.

### Gene enrichment and pathway analysis

#### Effect of sex on visceral adipose tissue gene expression pathways

**FIGURE 7:**
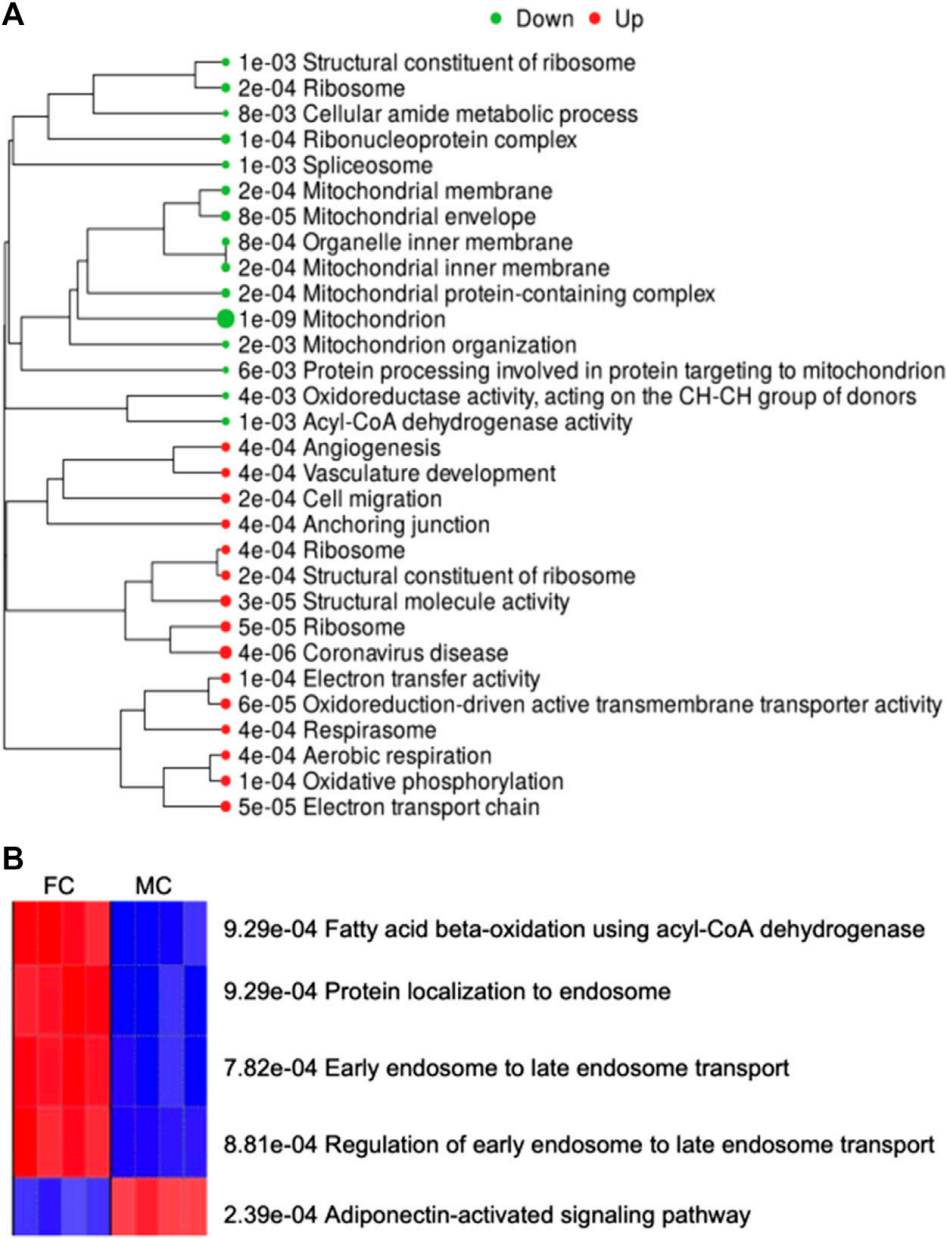
**(A)** Demonstrates enriched pathways in DEGs for genes altered on comparing male vs. female control fetuses. Green dots indicate downregulated pathways and red dots indicate upregulated pathways. The sizes of the dots correspond to the adjusted *p*-value. **(B)** Shows the GO Biological Process activated (Red) and suppressed (Blue) on comparing control males with control females.

On enrichment of the genes altered in males compared to females, the major network upregulated in males were those involved in neovascularization, and downregulated network involved mitochondrial function (Figure 7). On enrichment of differentially expressed genes in males compared to females, the Thermogenesis KEGG Pathway was downregulated, and the Metabolic and Steroid Biosynthesis KEGG pathways were upregulated (Supplementary Table S4). Furthermore, we examined the gene ontology (GO) biological process and observed that the adiponectin-activated signaling pathway was activated, and endosome-related pathways were suppressed in perirenal adipose tissue from control males as compared to those in control females (Figure 7B). The list of genes associated with these GO biological processes is provided in Supplementary Table S5.

#### Effect of growth restriction on gene expression pathways

**FIGURE 8:**
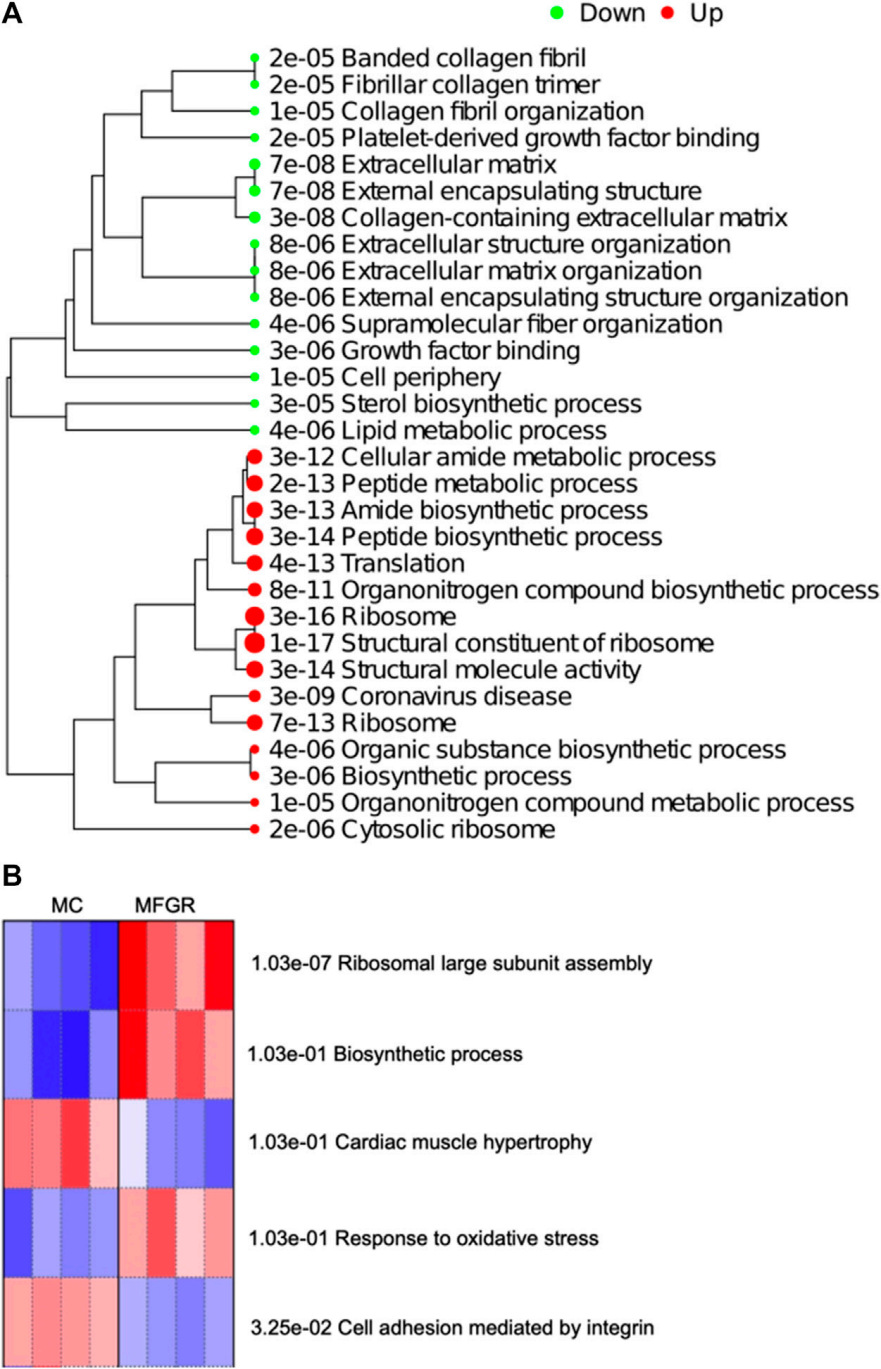
Demonstrate enriched pathways in DEGs for genes altered on comparing MFGR vs. MC. **(A)** Green dots indicate downregulated pathways and red dots indicate upregulated pathways. The sizes of the dots correspond to the adjusted *p*-value. **(B)** Shows the GO Biological Process activated (Red) and suppressed (Blue) on comparing control males with growth-restricted males.

The result demonstrates that female adipose tissue did not undergo major changes with growth restriction. Only 8 genes were altered in FGR females compared to control females, and they did not map to a common pathway. However, following fetal growth restriction, the translation and biosynthetic pathways were upregulated in males, and lipid/sterol pathways were downregulated (Figure 8). On enrichment of differentially expressed genes in MFGR compared to MC, the Steroid Biosynthesis KEGG Pathway was downregulated, and the PPAR Signaling KEGG pathway was upregulated (Supplementary Table S6; Supplementary Figure S2). On examination of the GO biological processes, we observed that in the perirenal adipose tissue from growth-restricted males, genes related to cell adhesion and hypertrophy were activated, and translation and oxidative stress-related genes were suppressed as compared to those in control males (Figure 8B). The list of genes associated with these GO biological processes is provided in Supplementary Table S7.

### Relative expression of preadipocytes, mature adipocytes, white adipocytes, and brown adipocytes markers in the four study groups

THY1 was used as preadipocyte markers; ADIPOQ and FABP4 were used as mature adipocyte markers UCP1 and CIDEA were used as brown adipose markers; and TCF21 and leptin were used as white adipose tissue markers (Table 1). Of note, leptin is also considered a mature adipose tissue marker. There was no change in any markers in females with growth restriction. In males with growth restriction, FABP4, a mature adipocyte marker, was significantly upregulated (>2.5 folds), and THY1, a preadipocyte marker, was downregulated (<0.5 fold). On examining the effect of sex on the markers, THY1 (preadipocyte marker) was downregulated in control males as compared to control females (>2 fold).

**TABLE 1:** Various adipocytes marker gene expression in the four study groups.

Gene id	Transcript id	Gene name	Transcript name	MFGR/MC fold change	MFGR/MC *p*-value	FFGR/FC fold change	FFGR/FC *p*-value	MC/FC fold change	MC/FC *p*-value
ENSOARG00020012680	ENSOART00020019420	UCP1	UCP1-201	1.174	0.920	−1.103	1.000	−1.240	0.654
ENSOARG00020017607	ENSOART00020027055	FABP4	FABP4-201	2.610	0.046	−2.276	0.647	-1.891	0.176
ENSOARG00020000398	ENSOART00020000499	ADIPOQ	ADIPOQ-201	−1.050	0.978	1.085	1.000	−1.023	0.952
ENSOARG00020023476	ENSOART00020036590	CIDEA	CIDEA 201	1.020	0.998	1.301	0.693	−1.091	0.656
ENSOARG00020016169	ENSOART00020024826	THY1	THY1 201	−2.208	0.188	−1.289	1.000	2.227	0.041
ENSOARG00020023476	ENSOART00020036602	CIDEA	CIDEA-202	−1.047	0.992	1.143	1.000	1.134	0.727
ENSOARG00020020602	ENSOART00020036602	TCF21	TCF21-201	−1.904	0.042	1.074	1.000	1.282	0.495
ENSOARG00020005038	ENSOART00020007691	ob	ob-201	−1.117	0.978	−2.050	0.896	1.182	0.811
ENSOARG00020018036	ENSOART00020027741	HOXC9	HOXC9 201	−1.190	0.677	−1.164	1.000	−2.081	0.000
ENSOARG00020016169	ENSOART00020024873	THY1	THY1-202	−2.396	0.016	−1.210	1.000	−4.989	0.000

### Relative expression of mitochondrial genes in the four study groups

To examine the mitochondrial density, we examined mitochondrial gene transcripts in the four groups. Males had significant upregulation of mitochondrial transcripts in perirenal adipose tissue (Table 2). Notably, there was no effect of growth restriction on mitochondrial transcripts in either male or females perirenal adipose tissue (Table 2).

**TABLE 2:** Mitochondrial gene expression in the four study groups.

Gene ID	Transcript ID	Transcript Nzme	MC/FC Fold Change	MC/FC P- Value	MFGR/MC Fold Change	MFGR/MC P-Value	FFGR/FC Fold Change	FFGR/FC P-Value
ENSOARG00020000007	ENSOART00020000007	ND1-201	1.07	0.87	-1.05	0.98	-1.01	1.00
ENSOARG00020000011	ENSOART00020000011	ND2-201	-1.26	0.34	-1.04	0.99	-1.04	1.00
ENSOARG00020000017	ENSOART00020000017	COX1-201	1.50	0.04	-1.07	0.96	-1.04	1.00
ENSOARG00020000020	ENSOART00020000020	COX2-201	1.43	0.11	1.06	0.98	-1.12	1.00
ENSOARG00020000022	ENSOART00020000022	ATP6-201	-1.76	0.00	-1.04	0.99	1.01	1.00
ENSOARG00020000023	ENSOART00020000023	COX3-201	1.79	0.00	-1.34	0.18	-1.11	1.00
ENSOARG00020000025	ENSOART00020000025	ND3-201	2.86	0.00	-1.09	0.96	1.02	1.00
ENSOARG00020000027	ENSOART00020000027	ND4-201	1.59	0.02	-1.12	0.88	1.02	1.00
ENSOARG00020000031	ENSOART00020000031	ND5-201	1.44	0.16	-1.28	0.63	-1.12	1.00
ENSOARG00020000032	ENSOART00020000032	ND6-201	13.89	0.00	-1.28	0.64	-1.06	1.00
ENSOARG00020000034	ENSOART00020000034	CYTB-201	1.37	0.16	1.07	0.97	-1.10	1.00

## Discussion

Fetal growth restriction is a known risk factor for obesity in adult life. The mechanisms of fetal programming of adult disease are still not well understood. In the present study, we demonstrate that ambient hyperthermia induces a significant growth restriction in fetuses. Although the overall perirenal weight was lower in growth-restricted fetuses, there was no significant difference with control fetuses following normalization to body weight. Several studies have shown that adipose tissue relative percentages are maintained or may be higher in an organism despite growth restriction (Gondret et al., 2006; Coupe et al., 2012; Lynegaard et al., 2020). In contrast, several studies have demonstrated that the percentage weight of several organs relative to the total body weight is significantly lower in growth-restricted fetuses (Man et al., 2016; Serpente et al., 2021). Notably, similar to adipose tissue, the brain is the other organ that shows relative sparing in weight reduction in response to growth restriction. Thus, active programming of the adipose tissue and protection indicates the importance of adipose tissue in organismal biology.

In recent years, adipose tissue has emerged as an important endocrine organ and is responsible for the secretion of several hormones (Coelho et al., 2013). In the present report, irrespective of growth restriction, we observed significant differences in gene expression in male versus female perirenal adipose tissue. In males, the neovascularization network was upregulated as compared to that in females. Similarly, the mitochondria-related genes were downregulated in males as compared to those in adipose tissue from female fetuses. In a recent report, it was demonstrated that with a high-fat diet (HFD), there was an increase in mitochondrial complexes in female mice as compared to those in male mice, and the male mice were more prone to obesity (MacCannell et al., 2021). Similarly, sexual dimorphism in adipose tissue has been demonstrated by others in response to a high-fat diet in mice (Chang et al., 2018) and baboons (Guo et al., 2013). Thus, the present study further demonstrates sexually dimorphic programming in response to growth restriction in sheep adipose tissue. Also, in control males, we observed that the genes involved in thermogenesis pathways are downregulated, and those involved in metabolic pathways, including steroid biosynthesis, are upregulated. Reduction in thermogenesis may be responsible for making FGR males more prone to obesity than control.

Also, fetal growth restriction has been implicated in the “programming” of obesity in later life (Ducsay et al., 2018; Goyal et al., 2019). In the present report, we observed sexually dimorphic alterations in the differentiation ability of preadipocytes with growth restriction. There was a significant reduction in cellular density only in male growth-restricted fetuses. In growth-restricted male fetuses, there was also a significant reduction in the preadipocyte differentiation potential to mature adipocytes compared to male controls. These changes were not observed in female fetuses. Moreover, previous reports demonstrate that female FGR babies are at significant risk for excessive fat deposition in adipose tissues and rapid catch-up growth in a sexually dimorphic manner in adult life (Hales and Barker, 2001; Tchoukalova et al., 2014; Dearden et al., 2018). Based on the findings from the present study, we speculate that changes in the male adipose gene expression may be playing a role sexually dimorphic risk for obesity in later life. However, further investigations and follow-ups in adult animals are required to gain a mechanistic understanding.

Similarly, in response to growth restriction, there was a significant change in the male transcriptome of perirenal adipose tissue, which was not observed in growth-restricted female fetuses. In growth-restricted male fetuses, the PPAR-gamma pathway was upregulated, and the PI3K-AKT pathway was downregulated with an associated increase in mature adipocyte marker and reduction in preadipocyte marker. Evidence supports that the PPAR-gamma pathway is involved in preadipocyte differentiation and maturation (Floyd and Stephens, 2012). Thus, it is possible that with growth restriction, male fetuses have a reduction in the preadipocyte population which may be the reason for reduced cell density in growth-restricted males.

A surprising finding of the present study was that transcriptomic profiles were unaffected by growth restriction in adipose tissue from female fetuses. In contrast, differences in male fetuses were observed at both transcriptomic and phenotypic levels. The significance of this sexually dimorphic programming and causal mechanisms warrants further investigation.

## Conclusion and perspective

The present study demonstrates sexual dimorphic programming of perirenal adipose tissue differentiation and gene expression. Our findings show that female fetuses are more resistant than male fetuses. Following growth restriction, the adipose tissue from male fetuses had lower cell density, upregulated mature adipocyte markers, and lower *in-vitro* differentiation potential. Further studies are needed to examine if lower preadipocyte differentiation potential in males leads to more fat deposition from early life. The study also demonstrates that the PI3K-AKT and PPAR-gamma pathways require further investigation and may be involved in growth restriction-induced fetal programming of adipose tissue.

## Data Availability

This study’s raw and analyzed datasets can be found online at the GEO Dataset Repository, accession no. GSE235935.

## Supplementary material

SUPPLEMENTARY FIGURE S1 Demonstrates box plot **(A)** and Density Plot **(B)** of Transformed RNAseq Data.

SUPPLEMENTARY FIGURE S2 Demonstrates upregulation of PPAR Signaling Pathway in MFGR compared to MC.

SUPPLEMENTARY TABLE S1 List of differentially expressed genes in MC compared to FC.

SUPPLEMENTARY TABLE S2 List of differentially expressed genes in MFGR compared to MC.

SUPPLEMENTARY TABLE S3 List of differentially expressed genes in FFGR compared to FC.

SUPPLEMENTARY TABLE S4 List of differentially expressed genes enriched in MC compared to FC following KEGG pathway analysis.

SUPPLEMENTARY TABLE S5 List of differentially expressed genes enriched in MC compared to FC following GO Biological Processes analysis.

SUPPLEMENTARY TABLE S6 List of differentially expressed genes enriched in MFGR compared to MC following KEGG pathway analysis.

SUPPLEMENTARY TABLE S7 List of differentially expressed genes enriched in MFGR compared to MC following GO Biological Processes analysis.
