# Supplementary figures and images for: Sexual dimorphic gene expression profile of perirenal adipose tissue in ovine fetuses with growth restriction
Source: Front Physiol. 2023 Aug 4;14:1179288. doi: 10.3389/fphys.2023.1179288 (PMC10437077; doi:10.3389/fphys.2023.1179288)

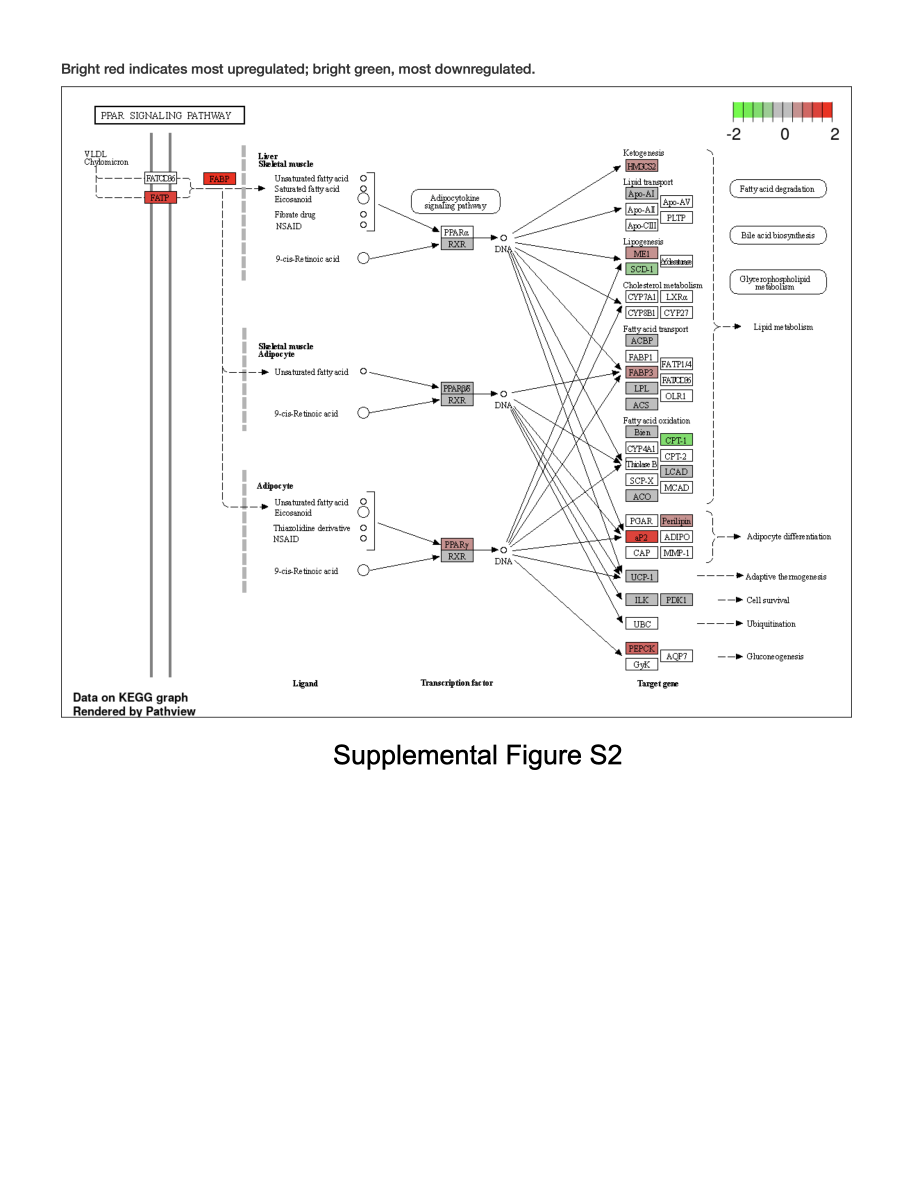

Supplement: Supplementary file 3 [file Image2.TIF]

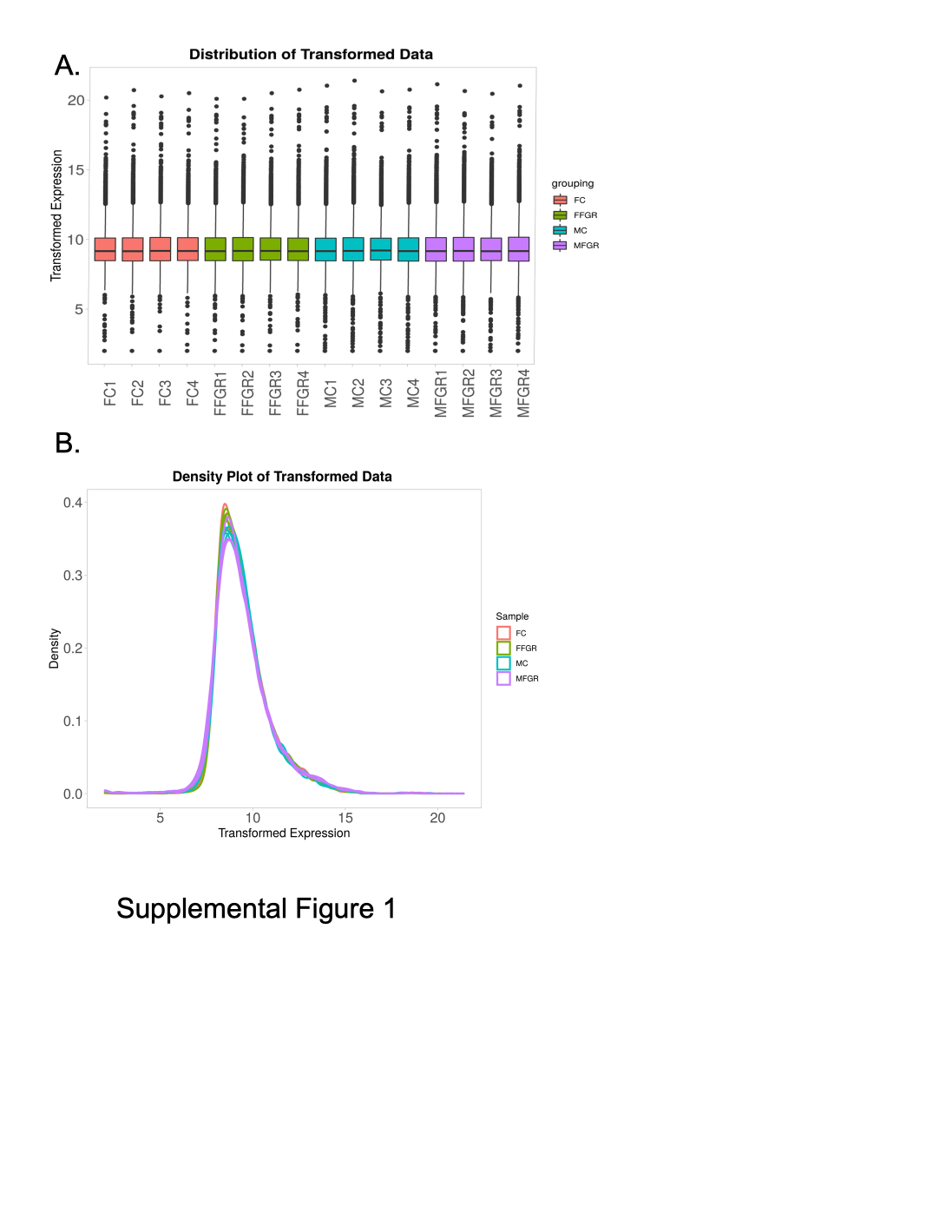

Supplement: Supplementary file 4 [file Image1.TIF]
